# Rapid Assay Diagnostic for Acute Stroke Recognition (RADAR): study protocol for a diagnostic accuracy study

**DOI:** 10.1136/bmjopen-2024-087130

**Published:** 2024-08-09

**Authors:** Lisa Shaw, David Burgess, Anand Dixit, Edoardo Gaude, Clare Lendrem, Graham McClelland, Phil White, Cameron Williams, Gewei Zhu, Christopher Price

**Affiliations:** 1 Stroke Research Group, Population Health Sciences Institute, Newcastle University, Newcastle upon Tyne, UK; 2 Service user representative, Stroke Research Group, Population Health Sciences Institute, Newcastle University, Newcastle upon Tyne, UK; 3 Newcastle upon Tyne Hospitals NHS Foundation Trust, Newcastle upon Tyne, UK; 4 Pockit Diagnostics Ltd trading as Upfront Diagnostics, Cambridge, UK; 5 NIHR Newcastle In Vitro Diagnostics Cooperative, Newcastle University and Newcastle upon Tyne Hospitals NHS Foundation Trust, Newcastle upon Tyne, UK; 6 Department of Nursing, Midwifery and Health, Faculty of Health and Life Sciences, Northumbria University, Newcastle upon Tyne, UK; 7 Stroke Research Group, Translational and Clinical Research Institute, Newcastle University, Newcastle upon Tyne, UK

**Keywords:** Triage, STROKE MEDICINE, Research Design

## Abstract

**Introduction:**

Large-vessel occlusion (LVO) stroke is effectively treated by time-critical thrombectomy, a highly specialised procedure only available in a limited number of centres. Many patients with suspected stroke are admitted to their nearest hospital and require transfer to access treatment, with resulting delays. This study is evaluating the accuracy of a new rapid portable test for LVO stroke which could be used in the future to select patients for direct admission to a thrombectomy centre.

**Methods and analysis:**

Rapid Assay Diagnostic for Acute Stroke Recognition (RADAR) is a prospective observational cohort study taking place in stroke units in England. Participants are adults with a new suspected stroke with at least one face, arm or speech (FAST) symptom(s) and known onset within 6 hours or last known to be well 6–24 hours ago. The index test (‘LVOne test’ (Upfront Diagnostics)), consists of two portable lateral flow assays which use fingerprick capillary blood to detect d-dimer and glial fibrillary acidic protein concentrations. Reference standards comprise independently adjudicated standard CT/MRI brain±CT/MR angiography with senior clinician opinion to establish: ischaemic stroke±LVO; intracerebral haemorrhage; transient ischaemic attack; stroke mimic. Analyses will report sensitivity, specificity and negative and positive predictive values for identification of LVO stroke. Powered using a primary analysis population (≥2 FAST symptoms and known onset within 6 hours), 276 participants will detect a test specificity of 92%. The broader total study population which allows evaluation of the test for milder symptoms and unknown onset times is estimated to be 552 participants.

**Ethics and dissemination:**

Ethical (North East—Newcastle & North Tyneside 2 Research Ethics Committee (reference: 23/NE/0043), Health Research Authority and participating National Health Service Trust approvals are granted. Consent is required for enrolment. Dissemination of results will include presentations at conferences, publication in journals and plain English summaries.

**Trial registration number:**

ISRCTN12414986.

**STRENGTHS AND LIMITATIONS OF THIS STUDY**

- This is a real-world diagnostic accuracy study involving participants representative of the prehospital suspected stroke population where the new test would be deployed in the future.
- The study has been designed to minimise selection bias and facilitate inclusion via the use of several invitation and consent options which cover incapacity, rapid discharge and death.
- A standardised approach to assign ‘gold-standard’ reference standard outcomes is used to support reproducibility of the data obtained.
- While hosting the study in hospitals may not truly reflect ambulance use of the test, this locality will provide robust data for diagnostic accuracy and it avoids the substantial challenges that arise when conducting research spanning both the prehospital and hospital settings.

## Introduction

 Stroke caused by cerebral ischaemia (85%) or haemorrhage (15%) affects around 13.7 million people across the world each year and causes a large burden of mortality and morbidity.^1^ In the UK alone, there are around 113 000 new strokes each year.^2^ Where ischaemic stroke is caused by occlusion of a large intracranial blood vessel (approximately 30% of ischaemic stroke),^3^ outcomes can be significantly improved by time-critical intra-arterial mechanical thrombectomy.^4 5^ This is a highly specialised procedure involving inserting an arterial catheter under X-ray guidance to extract the blood clot causing the stroke and can only be performed in appropriately resourced hospitals.

In the UK, there over 100 hospitals, which provide standard care for stroke patients (eg, thrombolysis) but only 24 of these centres are also able to undertake thrombectomy.^6 7^ As most patients with suspected stroke present to ambulance services which currently convey to the nearest hospital providing stroke care,^8^ onward transfer to a thrombectomy centre is required for many of the people suitable for treatment. National audit data from 2022/2023 demonstrate that it takes an average of over 3 hours from arrival at a first hospital to arrival at a thrombectomy centre.^9^ Similar delays to accessing thrombectomy are seen in other healthcare systems across the world.^10 11^

Direct admission of all suspected stroke patients to thrombectomy capable centres might decrease treatment delays but this is not typically considered a suitable service model for the UK.^6 7^ Capacity is limited but also many patients would not receive a diagnosis of large-vessel occlusion (LVO) stroke after investigation and therefore could be more appropriately treated in a local centre. This is because up to 40% of people suspected to be having a stroke by prehospital ambulance practitioners receive a non-stroke ‘mimic’ diagnosis after hospital assessment^12^ and only approximately 30% of ischaemic stroke are due to LVO.^3^

An alternative approach to facilitate timely access to thrombectomy treatment is to identify people likely to have LVO stroke prehospital and selectively redirect these individuals to a treatment centre.^13^ However, for this option to be feasible, a method of rapid and accurate LVO recognition in the prehospital setting is required. While there has been considerable interest in using prehospital symptom scale scores for this purpose, there are concerns about their real world accuracy when used in isolation.^13^

Recent work using biobank samples of venous blood drawn from suspected stroke patients in the emergency department within a mean of 2.6 hours from symptom onset indicated that combining blood concentrations of d-dimer and glial fibrillary acidic protein (GFAP) with clinical symptom scale scores (eg, Face Arm Speech Test (FAST), Field Assessment Stroke Triage for Emergency Destination (FAST-ED)) gave high area under the receiver operator curve (AUC) values for prediction of LVO stroke.^14^ When combining d-dimer and GFAP with the FAST, which is the symptom scale most commonly used by UK ambulance practitioners, the AUC was 0.93 with optimal specificity of 93% and sensitivity of 78%.^14^

These data led to the development of portable lateral flow assays to detect d-dimer and GFAP from fingerprick capillary blood, where results are available within 15 min (together named ‘LVOne test’: Pockit Diagnostics trading as Upfront Diagnostics). Using these lateral flow assays with existing biobank samples demonstrated that a specificity of 92% and sensitivity of 62% for LVO identification could be achieved from the combination of a positive d-dimer line, negative GFAP line and clinical symptom FAST score of 2 or 3 (unpublished data). The assays have been optimised for specificity because the future intended use of the measurements is prehospital triage for redirection whereby high specificity (ie, few false positives) is important to avoid inappropriate transfers.

As the next stage in the evaluation of the LVOne test, a diagnostic accuracy study using prospectively collected samples is being conducted. This manuscript describes the study protocol. Although the future intended purpose of the assays is prehospital use, this study is testing suspected stroke patients rapidly after hospital arrival. This provides a near identical population and avoids the substantial challenges which arise when conducting research spanning the prehospital/hospital setting such as the requirement for multiple testing devices (ie, for placement in all ambulances serving a region), training geographically dispersed ambulance personnel and linking prehospital and hospital patient data.

## Methods and analysis

### Study objectives

The primary objective is to determine the diagnostic accuracy of the LVOne test (using fingerprick capillary blood) for identification of LVO stroke.

Data-driven exploratory objectives may include examination of relationships between the diagnoses that constitute the suspected stroke population, blood biomarker values and key clinical variables (eg, stroke symptoms).

### Study design

The study design is a prospective observational cohort study.

### Study setting

The study is taking place within UK National Health Service (NHS) hospitals with established Hyperacute Stroke Units receiving direct ambulance admissions of patients with suspected stroke.

### Study population

Trained hospital staff (eg, research nurses, research trained doctors) undertake the LVOne test on patients who fulfil the following criteria:

#### Inclusion criteria

- Arrived at the study hospital by emergency ambulance.
- Aged 18 years or over.
- New acute stroke suspected by ambulance personnel before hospital arrival.
- At least one FAST symptom, ie, FAST score ≥1 (as determined by hospital staff).
- Stroke symptoms are known to have begun within the last 6 hours OR the patient was last known to be well between 6 and 24 hours ago (times determined by hospital staff).
- Blood sampling can be undertaken prior to any reperfusion treatment.
- Routine brain imaging is intended to be urgently performed.

#### Exclusion criteria

- Already assessed at another hospital and ambulance admission is a transfer for continuing care.
- Assigned a recent previous (within the last 4 weeks) diagnosis of deep vein thrombosis, pulmonary embolism, arterial embolism, stroke, transient ischaemic attack (TIA), long bone fracture, major trauma, any surgery under general anaesthesia (these diagnoses may result in elevated d-dimer).
- Suffered a recent previous (within the last 4 weeks) head injury which led to hospital attendance (this event may have resulted in elevated GFAP).
- Stroke symptoms are known to have begun greater than 6 hours ago.

### Participant identification and consent

Patients are assessed for study suitability immediately after arrival at hospital in parallel with their urgent clinical assessment. If a patient is considered to fulfil the eligibility criteria and staff trained about the study are in attendance, the LVOne test is undertaken following a short verbal explanation. A formal research consent process is not undertaken at this time to avoid delays to time-critical treatments.

Once emergency assessments and treatments are completed, all patients who have had the LVOne test are approached for study enrolment. Six different options for study invitation and consent are used to ensure that all tested patients can be potentially recruited. Similar methods have been used by the study investigator team in previous projects.^15 16^

#### Inpatient approach

##### Patients with mental capacity

For patients with capacity to consent to research, a trained member of the hospital staff will approach the patient to discuss the study, provide a standard patient information sheet and subsequently obtain consent in writing. If a potential participant is due to be discharged and wishes to have longer to consider the information before making a decision, a study postal consent form and prepaid reply envelope are issued. Telephone follow-up can be undertaken if the consent form is not returned, including the option to take consent verbally.

##### Patients with mild communication difficulties

For patients with mild communication difficulties due to the effects of stroke or a non-stroke mimic condition on the use and understanding of language (aphasia), a set of ‘easy access’ study documentation will be used. For people due to be discharged and requiring more time to consider participation, an early discharge ‘easy access’ postal consent form can be used, and/or the option to return later for a face-to-face consent discussion.

##### Patients lacking in mental capacity, personal consultee

It is anticipated that approximately one-third of study eligible patients will be unable to engage in an informed consent process due to the effects of stroke and non-stroke mimic conditions on communication and cognition. As exclusion of patients unable to engage with consent would reduce the clinical relevance of the study, enrolment via personal or nominated (professional) consultees is also used.

If a personal consultee can be identified, a discussion about the study is held, a personal consultee information sheet is provided and a consultee declaration form is subsequently completed. Initial discussion can be face to face or via the telephone according to availability, and the declaration form may be completed in person, via post or verbally.

##### Patients lacking in mental capacity, nominated consultee

For patients where a personal consultee cannot be identified, a nominated (professional) consultee is used with a nominated consultee information sheet and declaration form.

##### Early mortality

The early mortality rate following acute stroke is approximately 10% and some non-stroke mimic conditions are also associated with a high mortality, for example, severe infections. LVO stroke typically presents with more severe symptoms and some people with this type of stroke die rapidly following hospital admission. As the exclusion of patients who die soon after admission (and the LVOne test) would reduce the clinical relevance of this study, if a patient dies before consent can be obtained using one of the approaches described above, the local hospital investigator may complete an early mortality declaration form for study enrolment.

### Approach after discharge/transfer

Patients who are discharged from hospital before the consent process can be undertaken are invited to take part by post. An invitation letter, postal patient information sheet and postal consent form are used. Telephone follow-up can be undertaken if the consent form is not returned, including the option to take consent verbally.

### LVOne test (index test)

The LVOne test consists of two portable lateral flow immunoassays. Assay 1 detects d-dimer concentration and assay 2 detects GFAP concentration. When a sample of blood is added to an assay test strip, the target protein (ie, d-dimer or GFAP) is bound by an antibody which is conjugated to a nanoparticle. Following the addition of a reaction buffer, the target protein/antibody/nanoparticle complex flows through the test strip and binds with a second antibody forming a ‘sandwich’ which becomes visible as a coloured line according to the concentration of the target protein.

The d-dimer assay is semiquantitative where the intensity of the line colour is proportional to the concentration of d-dimer in the blood. The GFAP assay is qualitative and the presence of any visible line indicates that GFAP is present in the blood. A positive LVOne test is defined as a visible d-dimer line with an intensity score ≥4 out of 10 points established using a reference scorecard, and an absent GFAP line.

**Figure 1:**
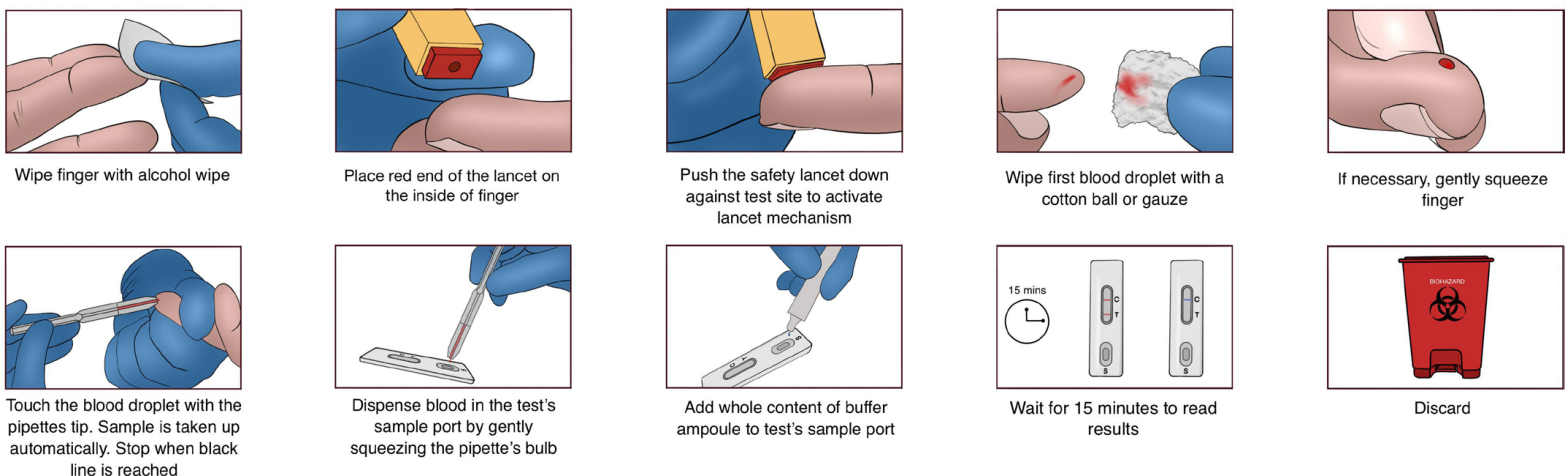
Performing the LVOne test.

The steps to perform the LVOne test are shown in figure 1.

### Reference standards (comparator)

Reference standards are required for key clinical outcome states which constitute the suspected stroke population: intracerebral haemorrhage, ischaemic stroke with LVO, ischaemic stroke without LVO, TIA and non-stroke mimic conditions.

Brain imaging tests are available which objectively confirm any intracerebral haemorrhage (CT) and ischaemic stroke with LVO (CTA/MRA). However, for ischaemic stroke without LVO, TIA or mimic conditions, no brain imaging or other diagnostic test alone exists, and for these cases, both the diagnosis assigned by a senior clinician at the participating hospitals and brain imaging findings will be reviewed to assign an outcome state. Full details are provided below and a similar approach has been used by the study investigator team in previous research.^16^

In keeping with routine practice for suspected stroke patients, it is anticipated that all participants in this study will undergo at least one brain imaging test. For the purposes of this study, all routine imaging performed within 72 hours of admission will be reviewed by an experienced consultant neuroradiologist blinded to clinical information and the LVOne test result.

#### Neuroimaging defined clinical outcome states

##### Intracerebral haemorrhage

Intracerebral haemorrhage will be the clinical outcome state assigned if a small (approximately <30 mL), medium (approximately 30–59 mL) or large (approximately ≥60 mL) volume haematoma is recorded during CT (and/or MRI) review by the blinded neuroradiologist.

##### Ischaemic stroke with LVO

Ischaemic stroke with LVO will be the clinical outcome state assigned if CT or MR angiography has been conducted and demonstrates reduced filling in any large branch of the anterior or posterior cerebral circulation. This will be recorded by the blinded neuroradiologist, with use of the 10-Point Clot Burden Score^17^ for the anterior circulation. Angiography is not routinely performed for all suspected stroke patients as clinical or other radiological investigations can exclude the need for this test, for example, plain CT has shown a haemorrhage or another radiological diagnosis for the acute symptoms such as a tumour.

### Other clinical outcome states

Because diagnoses recorded in medical records can vary according to the taxonomy used and the terminology preferred by individual clinicians (eg, chest infection may be used synonymously with pneumonia, bronchopneumonia and lower respiratory tract infection), local hospital clinicians will be asked to select a ‘definite’ or ‘probable’ primary clinical diagnosis according to a predefined framework.^15 16^ As diagnoses are sometimes uncertain for a time after admission to hospital, senior clinicians will be asked to provide a diagnosis assigned at 72 hours after hospital admission (or discharge/death if sooner).

#### Ischaemic stroke without LVO

Ischaemic stroke without LVO will be the clinical outcome assigned if the clinician considered the patient to have a ‘definite’ or ‘probable’ ischaemic stroke and CTA/MRA read by the blinded neuroradiologist confirms the absence of LVO.

#### Ischaemic stroke with unknown LVO status

There are likely to be participants with a clinical diagnosis of ischaemic stroke who have not undergone CTA/MRA for some reason. This may be due to the clinical situation (eg, agitation) or local hospital policies about access to this procedure. In these cases, the LVO status is unknown and a separate clinical outcome ‘ischaemic stroke with unknown LVO status’ will be assigned.

#### Transient Ischaemic Attack

Transient Ischaemic Attack will be the clinical outcome assigned if the clinician records any ‘definite’ or ‘probable’ TIA diagnosis using the predefined framework and brain imaging read by the blinded neuroradiologist does not contain findings which refute this opinion or indicates the presence of one of the other acute outcomes. Following resolution of any data discrepancies (see also below), if there remains any disagreement between blinded neuroradiological review of imaging performed and a clinical diagnosis of TIA, the imaging diagnosis will be used to determine the outcome.

#### Non-stroke mimic condition

A non-stroke mimic condition will be defined as present if the clinician records any ‘definite’ or ‘probable’ non-stroke/non-TIA diagnosis using the predefined framework and brain imaging read by the blinded neuroradiologist is compatible (eg, tumour) and does not contain findings which refute the opinion or indicates the presence of one of the other acute outcomes. Following resolution of any data discrepancies (see also below), if there remains any disagreement between blinded neuroradiological review of imaging performed and a clinical diagnosis of mimic condition, the imaging diagnosis will be used to determine the outcome.

### Assigning the clinical outcome state

The clinical outcome state will be assigned by a separate independent senior stroke clinician who will be blinded to LVOne test result. This clinician will review routine healthcare information collected for the study including the local clinician diagnosis and hospital routine imaging reports, and blinded neuroradiologist reports. A check for discrepancies in the information will first be performed (eg, routine hospital imaging reports are inconsistent with the blinded neuroradiology report, local clinician assigned diagnosis is inconsistent with imaging findings) and as required, data will be queried with the appropriate hospital team. Following any necessary resolution, a clinical outcome will subsequently be assigned according to the descriptions above. If any discrepancies cannot be satisfactorily addressed such that the independent clinician is able to assign a clinical outcome state, the case will be discussed by a Diagnostic Adjudication Committee. The committee will review anonymised clinical information plus the study blinded neuroradiologist report to agree on an outcome state. If the committee cannot reach a consensus, a decision that a clinical outcome state cannot be assigned will be taken.

The predefined diagnosis framework to be used by local hospital clinicians contains an ‘unclear’ option. If this option is selected, the case will also undergo discussion by the Diagnostic Adjudication Committee. If a consensus is reached about a primary diagnosis, a clinical outcome state will subsequently be assigned according to the descriptions above. If a consensus diagnosis cannot be agreed, it will not be possible to assign a clinical outcome state.

**Figure 2:**
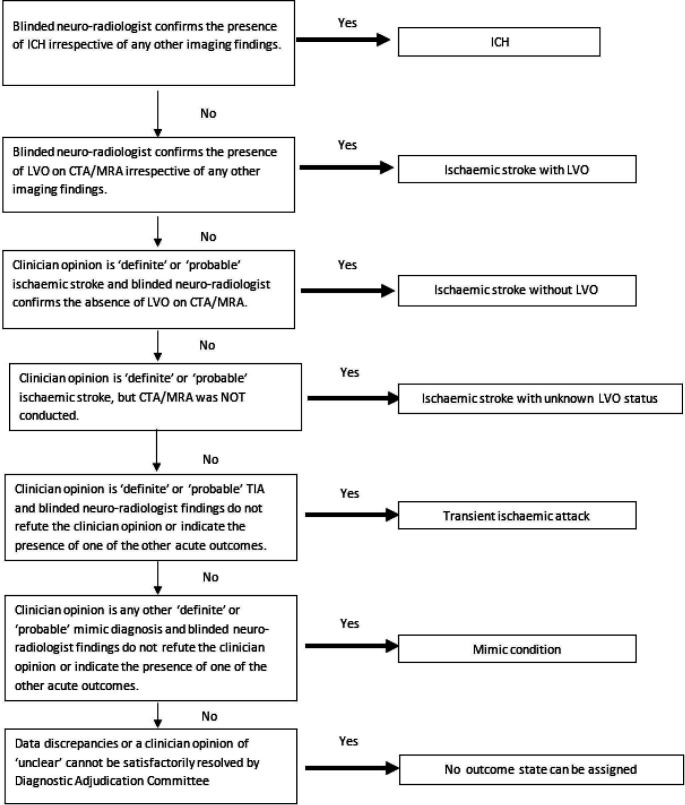
Decision tree for assigning the clinical outcome state. CTA, CT angiography; ICH, intracerebral haemorrhage; LVO, large-vessel occlusion; MRA, MR angiography; TIA, transient ischaemic attack.

Figure 2 shows a decision tree for assigning the clinical outcome state.

### Study data collection

For patients who give consent for enrolment in the study, data about the blood sampling processes, LVOne test lines status, safety data and routine healthcare information (eg, clinical symptoms, observations, a medical history, medication, laboratory blood test results, primary diagnosis and reperfusion treatments) will be collected. Copies of routine brain images will also be obtained for the neuroradiologist review.

Clinical data will be added to a secure online database managed by the study co-ordinating centre team. Copies of images will be provided on a CD or electronically according to local participating hospital preference. All data will be labelled with a unique study ID number only.

Where tested patients do not give consent to provide data for inclusion in the study, a record of which blood tests were undertaken with date/time and safety data only will be obtained.

### Blinding

The neuroradiologist(s) who reads brain images and the independent stroke clinician(s) who assigns the clinical outcome (reference standard), are blinded to the LVOne test results. Due to the nature of the LVOne test, hospital clinicians are not blinded to these results.

### Staff training and awareness

Study specific training is provided for staff at all participating hospitals by the investigator team.

### Study withdrawal

No specific withdrawal criteria have been preset. Participants may withdraw from the study at any time for any reason. Data collected prior to withdrawal will be used in the study analysis unless the patient or their representative requests that this should not be the case.

### Safety evaluation

This observational study involves fingerprick blood sampling which is a routine clinical procedure and the risks should be no greater than standard care. Any complications of the blood sampling processes are recorded on a paper study record which is completed when patients are having study blood tests undertaken. In addition, this data collection form requests information about any issues which arise during the use of the bespoke lateral flow equipment.

Should a medical event occur which is serious (results in death; is life-threatening; requires inpatient hospitalisation or prolongation of existing hospitalisation; results in persistent or significant disability or incapacity; consists of a congenital anomaly or birth defect and otherwise considered significant by the investigator) and is perceived to be related to the study test, a separate study serious adverse event form will be completed. All such events will be considered ‘unexpected’ and reported to relevant governance bodies.

### Sample size

As the intended purpose of the LVOne test is to ‘rule-in’ patients with LVO stroke, the study sample size calculation is based on demonstrating test specificity.

The sample size calculation is based on a ‘primary analysis population’ which consists of patients scoring 2 or 3 on the FAST clinical symptom scale who are also known to be within 6 hours of symptom onset. This is the population where previous data supports high test accuracy and is, therefore, the most likely future use case for the test, ie, the LVOne test would be deployed if an initial routine FAST test conducted by ambulance practitioners indicates a score of 2 or 3, and the patient is within 6 hours of symptom onset. Considering this primary analysis population, using an estimated prevalence of LVO of 32%, a minimum specificity of 80%, alpha=0.05 and beta=0.1, 123 participants are required to detect a specificity of 92% for LVO versus all other clinical outcomes in the study population.

Due to the nature of this clinical study being conducted in NHS hospitals, some enrolled participant’s data may not be complete for inclusion in the primary analysis, for example, it is not possible to assign a patient as LVO present or absent due to the nature of brain imaging undertaken (see also Reference standards section above). This lack of data is hard to estimate as individual hospital policies vary, however, based on assessment of likely available data from each proposed participating site, the primary analysis population sample size has been initially inflated to 276. During the study, there will be regular monitoring of the number of cases which can contribute to the primary analysis and fewer or additional participants will be sought accordingly.

The population being enrolled in the study is broader than the primary analysis population (as described in the Study population section above) and will include people suspected to be stroke by ambulance practitioners with a score of 1, 2 or 3 on the FAST clinical symptom scale and who are also either known to be within 6 hours of symptoms commencing or last known to be well between 6 and 24 hours ago. This broader study population has been chosen to allow for a number of other important analyses (see also Statistical analysis section below) including:

- Confirmation that the LVOne test results are most appropriately used in combination with a FAST score of 2 or 3, rather than FAST 1–3, ie, it is the combination of FAST 2/3, positive d-dimer and negative GFAP which indicates high diagnostic accuracy for LVO detection.
- Examination of whether or not the combination of FAST 2/3, d-dimer and GFAP is only highly accurate in patients presenting with symptoms known to have begun within 6 hours (data collected about the combination to date) or if it may also be sufficiently accurate to indicate LVO in people where symptom onset is unknown but they were last known to be well between 6 and 24 hours; this reflects increasing evidence of benefit from thrombectomy for people with unknown symptom onset plus favourable findings on advanced brain imaging techniques.

Patients fulfilling the study eligibility criteria but who are outside of the primary analysis population will be tested/consented to the study in parallel with those people who are within the primary analysis population, according to a presentation at participating hospitals and availability of study-trained staff. The number of ‘non-primary population’ participants who will be included in the study is dependent on the number likely to be tested/consented during the time taken to accrue the primary analysis population sample size. This is estimated to be 276 participants.

The overall study population sample size is estimated to be 552 participants.

### Statistical analysis

All statistical analyses will follow relevant reporting guidelines such as STARD (Standards for the Reporting of Diagnostic Accuracy Studies) 2015^18^ and TRIPOD (Transparent Reporting of a multivariable prediction model for Individual Prognosis or Diagnosis) 2015.^19^

All analyses populations and statistical plans will be fully described in a detailed statistical analysis plan, prepared and finalised prior to the end of data collection.

In addition to the primary analysis population described above (ie, FAST score 2 or 3 and known to be within 6 hours of symptom onset), other analysis populations will examine different combinations of FAST score and time after symptom onset.

According to analyses being conducted, some cases may be excluded with appropriate justification including:

- Patients who were enrolled but did not meet a study eligibility criterion and as such were enrolled in error, for example, it only becomes apparent after initial hospital assessment and testing that symptom onset was not within the required boundaries.
- Patients where a reference standard of ‘ischaemic stroke with unknown LVO status’ is assigned.

#### Primary analysis

Using the primary analysis population, the accuracy of the LVOne test will be reported as sensitivity, specificity, positive and negative predictive values with 95% CIs. A positive LVOne test is defined as a visible d-dimer line with an intensity score ≥4 out of 10 points (established using the reference scorecard), and an absent GFAP line.

#### Secondary analyses

Using other analysis populations, the accuracy of the LVOne test will be reported as sensitivity, specificity, positive and negative predictive values with 95% CIs. The analyses of these populations will be conducted and reported in the same way as the primary analysis.

#### Exploratory analyses

Acquired data will drive exploration of relationships which may include those between the diagnoses that constitute the suspected stroke population, LVOne test result, key clinical variables or additional stroke symptom scales (eg, FAST-ED). An alternative reference standard for LVO may be considered, for example, rather than only CT/MR Angiography confirmation, senior clinician assigned ischaemic stroke plus the presence of a dense middle cerebral artery sign on plain CT may be used to indicate LVO for patients who did not have angiography.

### Patient and public involvement

This research project involves a public representative as an investigator who was involved in the funding application, study design and contributes to ongoing study delivery. In addition, researchers regularly engage with two local PPI groups to discuss study design and progress.

## Ethics and dissemination

Ethical approval for this study was obtained from the North East—Newcastle & North Tyneside 2 Research Ethics Committee (reference: 23/NE/0043). Health Research Authority and participating NHS Trust approvals are also in place. Consent is required for enrolment and is obtained after the LVOne test and emergency care have been completed. Six different options are used for invitation and consent to ensure that all tested patients can be potentially recruited.

Dissemination of study results will include presentations at national and international conferences and events, publication in peer-reviewed journals, and plain English summaries for patient/public engagement activities.

The study is expected to report by the end of 2024.

## Discussion

Access to thrombectomy is currently inadequate in many healthcare systems. If a simple rapid prehospital test could identify probable LVO among the suspected stroke population and be used to direct admission to a thrombectomy provider, time to treatment could be decreased and patient outcomes improved. This ongoing study evaluating the performance of the LVOne test commenced in August 2023 and at the time of submission of this manuscript in March 2024 had enrolled 262 participants from five NHS hospitals.

The study has been designed to minimise bias by seeking participants representative of the prehospital suspected stroke population and facilitating inclusion using several options for invitation and consent (eg, options for incapacity, rapid discharge and death). The pragmatic approach of hosting the study in a hospital setting may not truly reflect ambulance deployment of the test but will provide robust data regarding diagnostic accuracy. The use of a standardised approach to assigning the ‘gold-standard’ reference diagnosis of LVO stroke as well as clear definitions for attribution of non-LVO states means that the results will have a high likelihood of being reproducible. The broad population being enrolled in the study will also provide information about how test performance is influenced by stroke symptom severity and time from symptom onset.
