# Supplementary material for: Rapid Assay Diagnostic for Acute Stroke Recognition (RADAR): study protocol for a diagnostic accuracy study
Source: BMJ Open. 2024 Aug 9;14(8):e087130. doi: 10.1136/bmjopen-2024-087130 (PMC11331886; doi:10.1136/bmjopen-2024-087130)
Supplement: Supplementary file 1 [file bmjopen-2024-087130.reviewer_comments.pdf]

PEER REVIEW HISTORY

BMJ Open publishes all reviews undertaken for accepted manuscripts. Reviewers are asked to complete a checklist review form (**http://bmjopen.bmj.com/site/about/resources/checklist.pdf**) and are provided with free text boxes to elaborate on their assessment. These free text comments are reproduced below.

ARTICLE DETAILS

**TITLE (PROVISIONAL)**	Rapid Assay Diagnostic for Acute stroke Recognition (RADAR): study protocol for a diagnostic accuracy study
**AUTHORS**	Shaw, Lisa; Burgess, David; Dixit, Anand; Gaude, Edoardo; Lendrem, Clare; McClelland, Graham; White, Phil; Williams, Cameron; Zhu, Gewei; Price, Christopher

This article was not externally reviewed at BMJ Open. Protocols that have been independently assessed prior to submission to BMJ Open are usually fast-tracked to publication on the grounds that further substantial changes will not be possible. This independent assessment will usually be external, independent review for both a substantial grant award from a non-commercial or government funder and ethics approval.
